# Fabrication and performance evaluation of nZVI integrated PVDF nanocomposite membranes for the remediation of lead contaminated wastewater

**DOI:** 10.1186/s11671-026-04776-3

**Published:** 2026-07-10

**Authors:** Md. Rezaur Rahman, Murtala Namakka, Khairul Anwar Bin Mohamed Said, Ismail Rahman

**Affiliations:** 1 Department of Chemical Engineering and Energy Sustainability, Universiti Malaysia Sarawak, 94300 Kota Samarahan, Sarawak Malaysia; 2 Department of Chemical Engineering, Ahmadu Bello University, Zaria, 800001 Nigeria; 3 Institute of Environmental Radioactivity, Fukushima University, 1 Kanayagawa, Fukushima City, 960-1296 Japan

**Keywords:** Nanocomposite membrane, Hydrophilicity, Nanoparticle, Zero-valent iron, Lead removal, Wastewater treatment

## Abstract

Water contamination with potentially toxic elements (PTEs) poses a critical challenge to environmental integrity and public health. While wastewater treatment technologies have advanced, further innovations are required to enhance the removal efficiency and flux performance of nanocomposite membranes for lead (Pb^2+^), a major PTE, in the wastewater matrix. In this study, nanoscale zero-valent iron (nZVI) particles were synthesized via chemical reduction and subsequently incorporated into polyvinylidene fluoride (PVDF) membranes via a phase-inversion technique. Six distinct membrane configurations (Y0–Y5) were developed by varying the nZVI loading. The physicochemical properties and performance of these PVDF-nZVI membranes were investigated using FESEM, FTIR, EDX, XRD, XPS, TGA, water contact angle measurements, solvent content analysis, pure water flux, and Pb^2+^ removal efficiency. The influence of varying Pb^2+^ concentrations on PVDF-nZVI membrane performance was investigated. The removal efficiencies of the PVDF-nZVI membranes consistently exceeded 75% across all configurations, with the optimal membrane (Y3) achieving 96% Pb^2+^ removal while maintaining stable hydraulic properties throughout a 50-min filtration operation. These findings demonstrate the potential of PVDF-nZVI nanocomposite membranes for effective lead (Pb^2+^) remediation and their applicability in advanced wastewater treatment technologies.

## Introduction

The contamination of wastewater with potentially toxic elements (PTEs), primarily from industrial discharges, poses a severe threat to ecological stability and human health. Among these pollutants, lead (Pb^2+^) is particularly concerning due to its high toxicity and persistence in the environment. Consequently, the development of advanced remediation materials capable of efficiently removing Pb^2+^ is essential to mitigate health risks and prevent long-term environmental degradation [14, 36].

Conventional remediation strategies, including chemical precipitation, ion exchange, and electrochemical methods, are widely utilized but face significant intrinsic limitations. Ion exchange processes often suffer from poor selectivity and high resin production costs, while electrochemical methods are hampered by the formation of passive, non-conductive layers that reduce efficiency over time. Advanced oxidation processes (AOPs) have been explored to improve selectivity and sorption capacity [32], yet they are often constrained by prolonged operational times and excessive sludge generation [16]. As a result, sorption remains the most practical and widely adopted strategy [22]. However, its efficacy is strictly governed by the catalytic activity and stability of the sorbent material.

Nanoscale zero-valent iron (nZVI) has emerged as a promising candidate for PTE remediation due to its exceptional reactivity, high reductive capacity, and ability to act as a large surface area electron donor [76]. However, nZVI-based sorbents often face challenges related to particle agglomeration and unstable sorption efficiencies [29]. To overcome these issues, incorporating nZVI into a polymer matrix offers a viable solution. Polyvinylidene fluoride (PVDF) is a preferred membrane material due to its outstanding mechanical strength [33, 45, 63, 64], chemical resistance, and thermal stability. However, pristin PVDF membranes are inherently hydrophobic, which can limit water permeability and separation efficiency. The incorporation of hydrophilic nanoparticles, such as nZVI, into the PVDF matrix can effectively modify the membrane surface [54], enhancing both hydrophilicity and contaminant rejection performance [68]. However, recent studies show that contaminants loading and long term filtaration operations can influence the susceptibility of nZVI based nancomposite membrane to significant fouling. Chen et al., [12] examined fouling of nZVI-PVDF membranes by xanthan gum (XG) and humic acid (HA) mixtures using ATR-IR-MCR-ALS. Fouling severity was found to be highest at 25% XG / 75% HA and lowest at 60% XG / 40% HA, with spectral analysis revealing that the dominant foulant shifted from XG to HA as the XG fraction increased.The presence of these foulants influence membrane matrix and decrease overall membrane performance. Li et al., utilized a simple filter-press coating method to fabricate nZVI-PVDF membranes for 2-chlorophenol (2-cp) removal. The membranes achieved 56.06–74.62% removal efficiency [39, 40]. These limitations viz. low pollutant degradtion and fouling susceptivity can be associated with the physicochemical properties and overral chemical stability of the nZVI material, since synthesis method and other factors such as pH are reported to influence nZVI chemical reactivity and stability [46, 48, 49].

In this study, nZVI nanoparticles were synthesized via a chemical reduction method to produce sorbents with distinct physicochemical properties suitable for membrane integration. PVDF-nZVI nanocomposite membranes were subsequently fabricated using the non-solvent induced phase separation technique. Varying loadings of nZVI (0.10–0.30 g) were incorporated to create membrane configurations designated Y1–Y5, with a pristin PVDF membrane (Y0) serving as the control. The study comprehensively characterizes the synthesized nanoparticles and membranes and evaluates their performance in terms of hydrophilicity, solvent uptake, pure water flux, and Pb^2+^ remediation at various concentrations.

## Experimental section

### Materials

All chemical reagents utilized were of analytical grade and used without further purification. Sodium borohydride (NaBH_4_), ferrous sulfate heptahydrate (FeSO_4_·7H_2_O), ethanol (CH_3_CH_2_OH), methanol (CH_3_OH), sodium hydroxide (NaOH), and hydrochloric acid (HCl) were procured from Sigma-Aldrich Sdn. Bhd. (Petaling Jaya, Malaysia). For the membrane matrix, polyvinylidene fluoride (PVDF) pellets (CAS No: 24937-79-9, *M*_w_ 534,000 g mol^–1^) were selected for their chemical resistance. N-Methyl-2-pyrrolidone (NMP) was used as the solvent to ensure homogeneous dispersion of the nZVI nanoparticles. All aqueous solutions were prepared using ultrapure water, and industrial-grade nitrogen (N_2_) was used to provide an inert atmosphere during experiments.

### Synthesis of nZVI nanoparticles

nZVI nanoparticles were synthesized via the liquid-phase reduction of FeSO_4_·7H_2_O by NaBH_4_, following the protocol described elsewhere [80, 81] with minor modifications. The ferrous solution was prepared by dissolving 17.80 g of FeSO_4_·7H_2_O in 300 mL of ultrapure water under continuous mechanical stirring. Separately, a reducing solution was prepared by dissolving 1.60 g of NaBH_4_ in 50 mL of ultrapure water. The reduction reaction was initiated by adding the NaBH_4_ solution dropwise to the FeSO_4_ solution. The reaction was allowed to proceed for 25 min at constant stiring (200 rpm) under nitrogen condition, during which the solution turned black. The cessation of hydrogen gas evolution indicated the completeness of the reduction of Fe^2+^ to Fe^0^ (Eqs. 1–3).

1 $${\mathrm{F}\mathrm{e}\mathrm{S}\mathrm{O}}_{4}\cdot {7\mathrm{H}}_{2}\mathrm{O}+2{\mathrm{N}\mathrm{a}\mathrm{B}\mathrm{H}}_{4}\to {\mathrm{F}\mathrm{e}}^{0}+{\mathrm{N}\mathrm{a}\mathrm{S}\mathrm{O}}_{4}+{7\mathrm{H}}_{2}\mathrm{O}+{2\mathrm{B}(\mathrm{O}\mathrm{H})}_{3}$$

2 $${\mathrm{F}\mathrm{e}}^{2+}+2{\mathrm{B}\mathrm{H}}_{4}^{-}+{6\mathrm{H}}_{2}\mathrm{O}\to {\mathrm{F}\mathrm{e}}^{0}+{2\mathrm{B}(\mathrm{O}\mathrm{H})}_{3}+{7\mathrm{H}}_{2}\uparrow$$

3 $${\mathrm{F}\mathrm{e}}^{0}\to {\mathrm{F}\mathrm{e}}^{2+}+2{\mathrm{e}}^{-}$$

The resulting black precipitate was collected via centrifugation for 5 min and subsequently dried in a tube furnace at 85 °C for 8 h under a nitrogen atmosphere to prevent oxidation.

### Fabrication of nZVI nanocomposite membranes

The nanocomposite membranes were fabricated using the phase inversion technique. A homogeneous dope solution was prepared by dissolving PVDF pellets (14% w/v) in NMP. nZVI nanoparticles were added to the solution at varying loadings: 0.10 g (Y1), 0.15 g (Y2), 0.20 g (Y3), 0.25 g (Y4), and 0.30 g (Y5). A control membrane (Y0) was prepared without nZVI. The mixtures were stirred vigorously at 350 rpm and heated to 85 °C for 24 h to ensure complete dissolution and uniform particle distribution.

To prevent defect formation, the dope solutions were degassed at 50 °C for 1 h to remove entrapped air bubbles. The bubble-free solution was then cast onto a glass substrate using a doctor’s blade to ensure uniform thickness. The cast film was immediately immersed in a deionized water coagulation bath at room temperature to initiate non-solvent induced phase separation. The nascent membranes were transferred to a fresh water bath and stored for 12 h to ensure complete phase inversion and the removal of residual NMP solvent. Finally, the membranes were stored at 4 °C before analysis. Table 1 summarizes the membrane compositions.

**Table 1:** Coding designation and composition of the fabricated PVDF-nZVI nanocomposite membranes

Code	PVDF (wt%)	Concentration of nZV (g)	NMP Solvent (v%)	Mass ratio nZVI/PVDF (g/g)
Y0	14	0	86	0
Y1	14	0.10	86	0.00714
Y2	14	0.15	86	0.0107
Y3	14	0.20	86	0.0143
Y4	14	0.25	86	0.0179
Y5	14	0.30	86	0.0214

### Characterizations

The physicochemical properties of the nZVI nanoparticles and the composite membranes were analyzed using a suite of advanced characterization techniques. Functional groups were identified using a Shimadzu IRAffinity-1 spectrometer in attenuated total reflectance (ATR) mode, with spectra recorded from 4000 to 400 cm^–1^ at a resolution of 4 cm^–1^ over 32 scans. Surface morphology and elemental composition were examined using a Hitachi S-4700 field-emission scanning electron microscope (FESEM-EDS) at an accelerating voltage of 20 kV, with images captured at magnifications of 10,000× and 50,000× . Thermal stability was assessed by thermogravimetric analysis (TGA) under a nitrogen atmosphere, monitoring key degradation points, including the onset at 323.7 °C and the inflection point at 372.9 °C. Chemical bonding states were probed using an Alpha-K XPS spectrometer utilizing monochromatic Al Kα radiation, with a pass energy resolution set between 11.75 and 23.5 eV. Furthermore, the crystalline structure was determined using a Rigaku SmartLab diffractometer (XRD) scanning from 0° to 80° (2θ), with crystallinity parameters calculated using the Scherrer equation (Eqs. 4–6) (*A*_c_, Area of all the crystalline peak; *A*_m_, Area of amorphous peaks; *A*_t_, Total area of all peaks; *k*, Scherrer constant = 0.94; λ(nm), X-ray wavelength = 1.5418; β(radians), the full width at half maximum (FWHM);

θ(degree), Bragg angle).

4 $$\text{Crystalline\, index} (\%) = \frac{{A}_{\mathrm{c}}}{{A}_{\mathrm{t}}} \times 100$$

5 $${A}_{\mathrm{t}} = {A}_{\mathrm{C}} +{A}_{\mathrm{m}}$$

6 $$D= \frac{k\lambda }{\beta cos\theta }$$

### Membrane performance evaluation

#### Physical and surface properties

Porosity of the nZVI nanocomposite membranes was determined by the gravimetric method [57], immersing samples in deionized water for 24 h and calculating porosity ($\varepsilon$) based on the wet (${W}_{\mathrm{a}}$) and dry (${W}_{\mathrm{b}}$) weights of dry membrane (g) (Eq. 7) (${\rho}_{w}$_,_ density of water, 0.998 g/cm^3^; ${\rho}_{\mathrm{P}\mathrm{V}\mathrm{D}\mathrm{F}}$_,_ density of PVDF polymer, 1.740 g/cm^3^).

7 $$\varepsilon \left(\mathrm{\%}\right)= \frac{\left(\frac{{W}_{\mathrm{a}}- {W}_{\mathrm{b}}}{{\rho}_{\mathrm{w}}}\right)}{\left(\frac{{W}_{\mathrm{a}}- {W}_{\mathrm{b}}}{{\rho}_{\mathrm{w}}}\right)+\left(\frac{{W}_{\mathrm{b}}}{{\rho}_{\mathrm{P}\mathrm{V}\mathrm{D}\mathrm{F}}}\right)}\times 100$$

Surface wettability of the fabricated nZVI membranes was quantified by static water contact angle measurements using an Ossila goniometer via the sessile drop method with ultrapure water as the probe liquid [24]. To account for surface heterogeneity, measurements were taken at multiple random locations on each sample. The temporal stability of the wettability was also evaluated. The final reported value for each membrane is the arithmetic mean of these replicates.

The solvent uptake and swelling of the nZVI nanocomposite membrane were tested for stability and affinity to water, ethanol, and methanol. Following established protocols [26], 1 cm^2^ membrane samples were immersed in each solvent for 24 h. Swelling was measured as the mass difference between wet (${W}_{\mathrm{s}}$) and dry (${W}_{\mathrm{d}}$) states, and equilibrium solvent content (*S*_c_, %) was calculated accordingly (Eq. 8).

8 $${S}_{\mathrm{c}} = \frac{{W}_{\mathrm{s}}- {W}_{\mathrm{d}}}{{W}_{\mathrm{s}}}\times 100$$

#### Hydraulic performance analysis

Hydraulic performance was evaluated using a dead-end filtration cell (effective diameter 4.4 cm). The system was primed with 2.5 L of pure water, pre-compacted at 2.0 bar for 30 min, and operated at 1.0 bar for 65 min to determine pure water flux (${J}_{w}$) (Eq. 9) (*Q*, permeate volume (L), *A*, membrane area (m^2^), and $\Delta T$, time (h).

9 $${J}_{w}= \frac{Q}{A \Delta T}$$

#### Analysis of lead (Pb^2+^) removal

Lead removal was assessed using aqueous Pb^2+^ solutions. A preliminary screening was conducted using a 10 ppm Pb^2+^ solution to determine the optimal membrane configuration. The optimized membrane was subsequently tested against higher concentrations (20, 30 and 40 ppm). Before filtration, membranes were compacted at 2 bar for 20 min, followed by a 10-min stabilization in the metal solution. Pb^2+^ concentrations in the feed (*C*_f_) and permeate (*C*_p_) were analyzed using Atomic Absorption Spectroscopy (AAS) to calculate the removal rate (*R*%) (Eq. 10).

10 $$R \left(\mathrm{\%}\right)=\left[1- \left(\frac{{C}_{\mathrm{p}}}{{C}_{f}}\right)\right]\times 100$$

## Results and discussion

### Characterization of synthesized nZVI nanoparticles

**Fig. 1:**
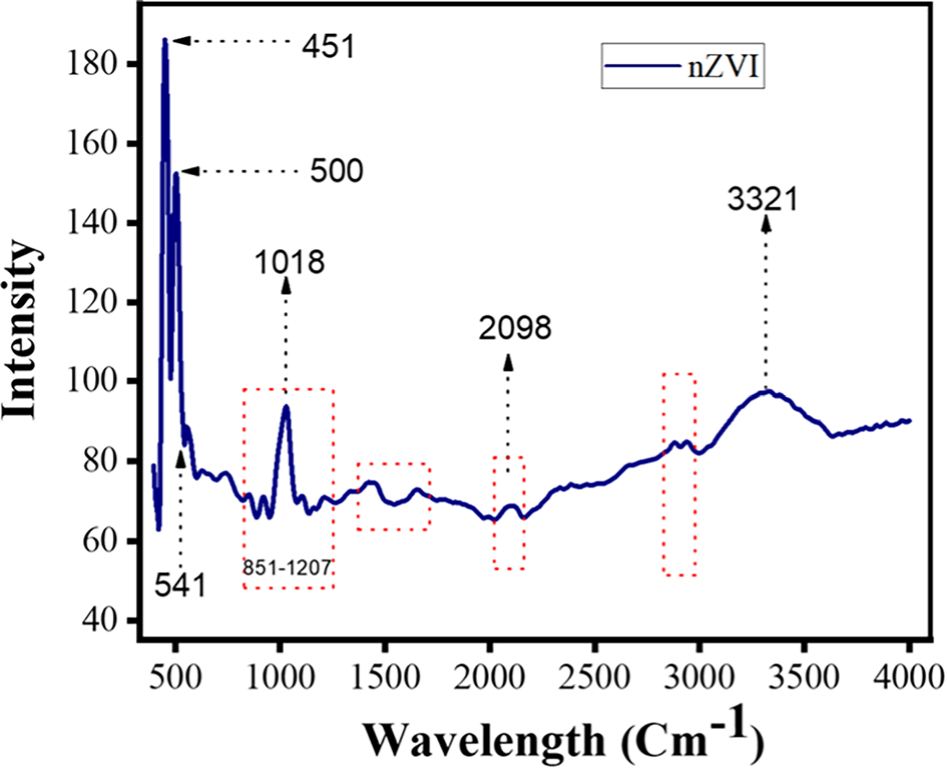
FTIR spectra of the synthesized nZVI nanoparticles showing characteristic functional groups

FTIR analysis identified key functional groups in the synthesized nZVI nanoparticles (Fig. 1). A broad band at 3321 cm^–1^ indicates O–H stretching, which supports particle stability in water. Peaks at 1590.78, 851, and 2098 cm^–1^ represent C=O stretching, C–H bending, and O–H bonds, suggesting adsorption of organics and oxidized species formation on the nZVI surface [46, 47, 78, 79]. Fe–O metal–oxygen bonds were detected between 451 and 541 cm^–1^, essential for nZVI structure [62], [74], while vibration intensities confirmed surface hydroxyl groups and their role in adsorption and catalysis, with Fe–O vibrations at 500 cm^–1^ and O–H related to Fe–OH at 600 cm^–1^.

**Fig. 2:**
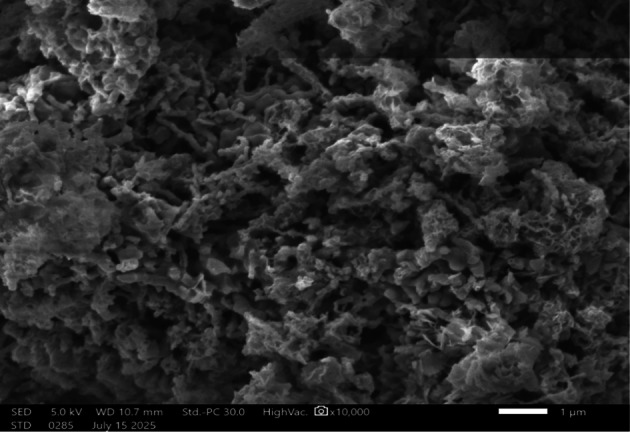
FESEM micrographs of the synthesized nZVI nanoparticles at 10,000 × magnification, showing particle morphology and concentration dependence tendencies

The morphological structure of nZVI nanoparticles, analyzed via FESEM, at 10,000 × magnification, is shown in Fig. 2. While the nZVI material is homogeneous mainly, some irregularly shaped particles are present, a feature consistent with mesopores observed in prior studies [39, 82].

**Fig. 3:**
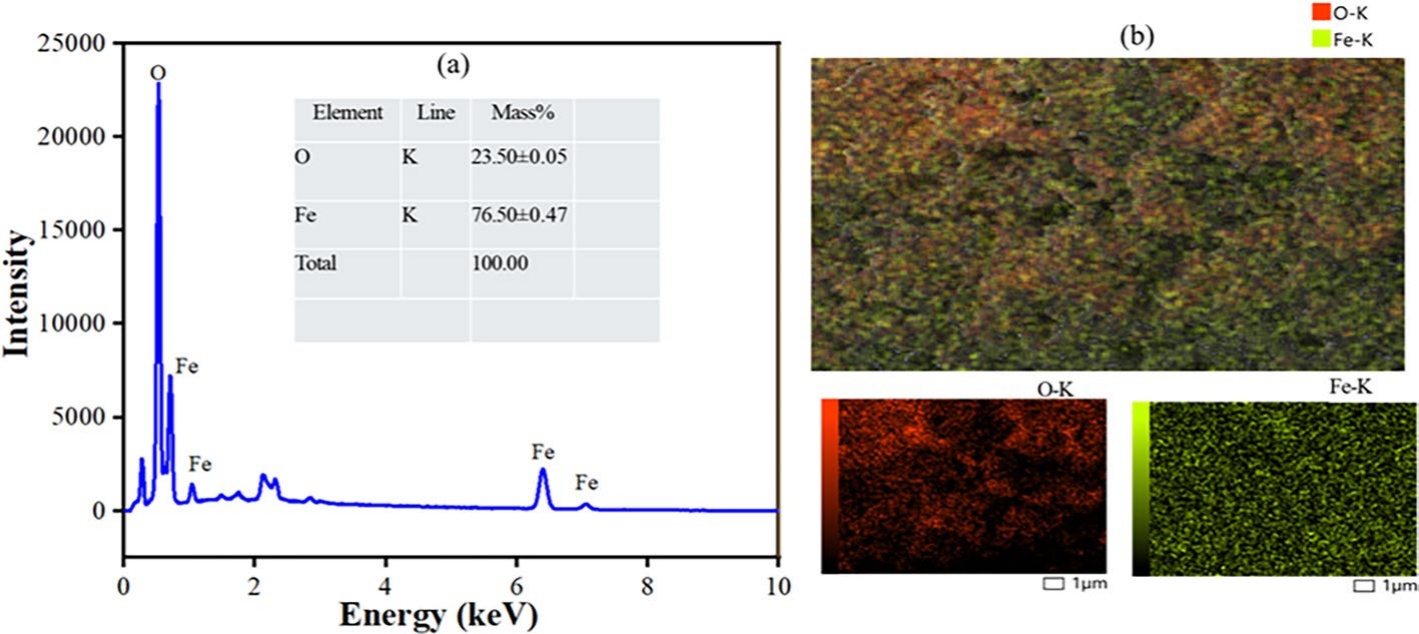
EDX spectrum and elemental composition of the synthesized nZVI nanoparticles

EDS analysis (Fig. 3) confirmed a high iron mass percentage of 76%, validating the Fe^0^ core, while the presence of oxygen confirms the core–shell structure with a surface Fe–O layer. These features result from interfacial interactions and the oxidation potential of nZVI, which affect its chemical stability and depend significantly on reaction pH [46, 47, 47].

**Fig. 4:**
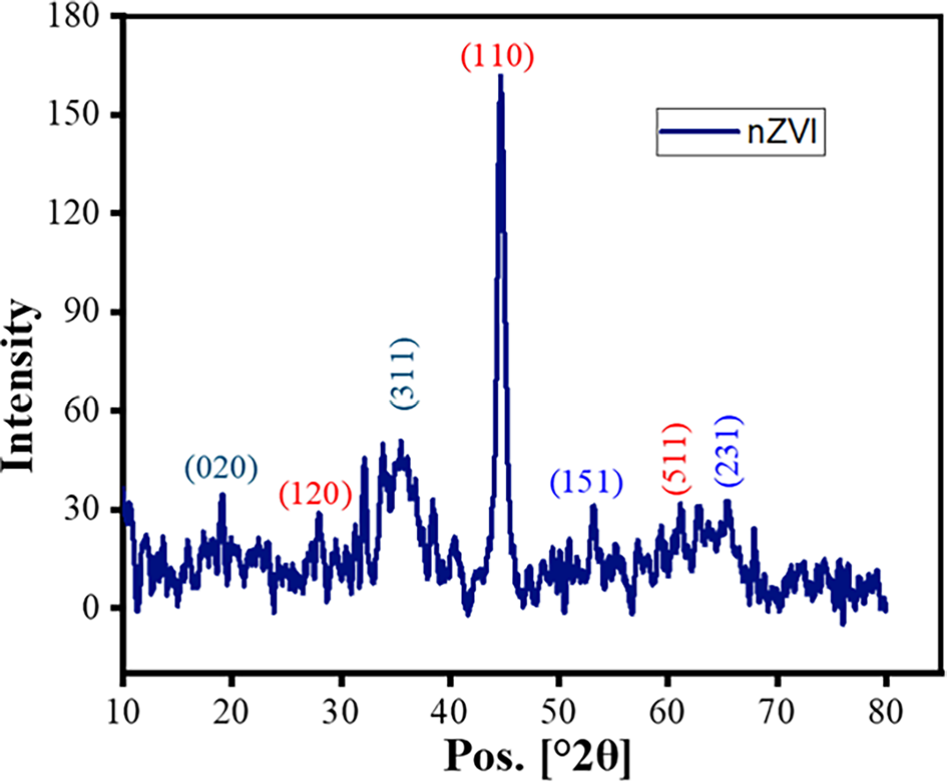
X-ray diffraction (XRD) pattern of the nZVI nanoparticles showing characteristic Fe^0^ and Fe–O peaks

X-ray diffraction (XRD) patterns (Fig. 4, Table 2) further corroborated the presence of metallic zero-valent iron (Fe^0^) [15, 23, 42, 65]. A distinctive magnetic Fe^0^ peak appears at 45° (110, JCPDS 06-0696), characteristic of nZVI materials [61]. Minor broad peaks at 64° and 66° (iron 511 and 231 planes) correspond to oxidized iron species, and a reflection at 37.4° suggests the formation of iron oxide shells (e.g., Fe_3_O_4_, γ-Fe_2_O_3_), which stabilize the reactive core [2, 4].

**Table 2:** XRD reflection indices and crystallographic planes of the synthesized nZVI nanoparticles

Phase	2θ (˚)	Crystal plane (hkl)	JCPDS Card	Refs
Fe	44.7	(110)	06–0696	[61]
Fe	65.1	(200)	06–0696	[13]

**Fig. 5:**
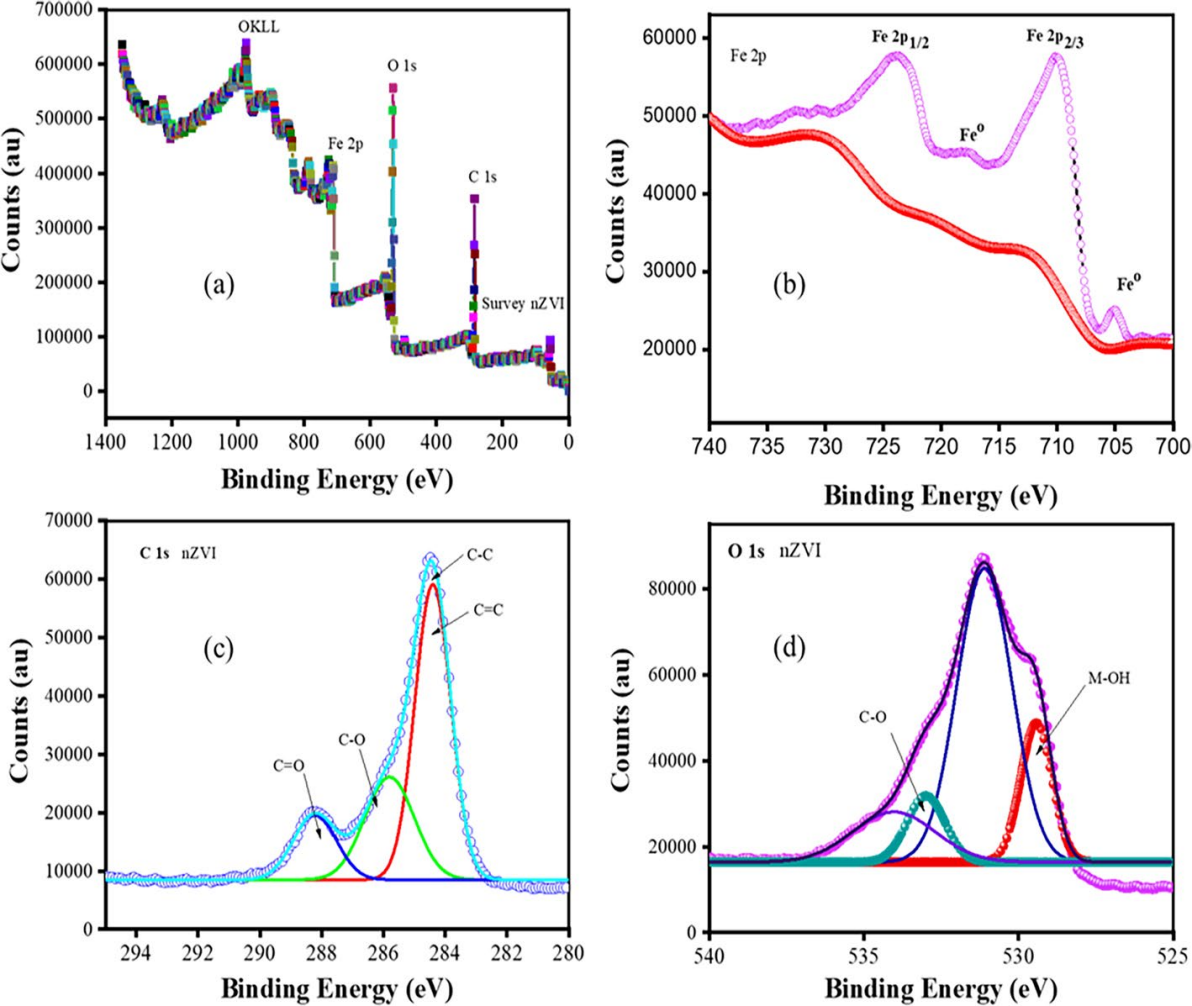
XPS analysis of nZVI nanoparticles: **a** Full survey spectrum; **b** Fe 2p detailed spectrum; **c** C 1 s detailed spectrum; and **d** O 1 s detailed spectrum

XPS analysis (Fig. 5) provided detailed insight into the chemical states of nZVI nanoparticles. The full survey confirms Fe^0^ presence (Fig. 5a), while Fig. 5b identifies Fe 2p_3/2_ and Fe 2p_½_ spin–orbit splitting peaks, with minor peaks at 705.6 eV and 718.6 eV, consistent with previous studies [40, 41, 67]. Peaks at 711 eV and 725.5 eV indicate oxidized iron species (Fe^2+^, Fe^3+^) and possible formation of Fe–OOH and Fe_3_O_4_ [4], confirming nZVI’s core–shell structure. C 1 s spectra reveal four prominent peaks at 284.8 eV (C–C, C–H from hydrocarbons) [19], 286.0 eV (C–O/C–OH), 288.5 eV (C=O/O–C=O) [10, 37] (Scientific, n.d.; X-ray, n.d.), and 283.0–284.0 eV (metal carbides, especially Fe–C) [20]. The O 1 s spectrum shows a strong peak at 528.1 eV (lattice oxygen) and another at 532.5 eV (M–O-H) [66], supporting surface hydroxyl groups on Fe–OOH or Fe_2_O_3_ [9]. These characteristics are important for material catalytic activity and structural integrity.

**Fig. 6:**
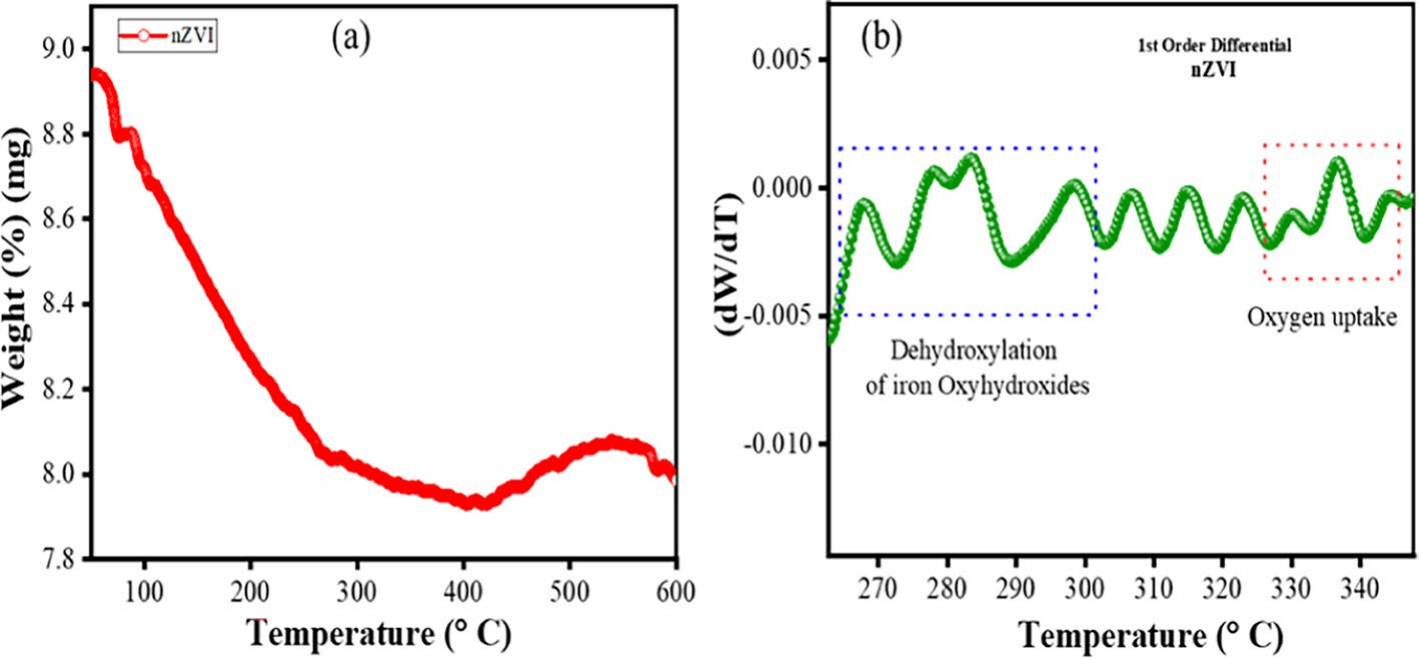
Thermal analysis of nZVI nanoparticles: **a** Thermogravimetric Analysis (TGA) curve; and **b** First-order derivative weight loss curve

Thermogravimetric analysis (TGA) (Fig. 6) revealed a cumulative weight loss of 38% of the nZVI composite throughout the observed temperature range. The initial phase of degradation at 150 °C corresponds to a weight reduction of 5 to 8%, primarily attributable to the release of physically adsorbed water and residual solvent molecules [47, 75]. Between 150 and 400 °C, further weight loss is attributed to the decomposition of surface organic species—such as –OH groups on the nanocomposite surface [34]. Subsequently, a weight gain observed from approximately 420 °C results from oxidation of the nZVI core leading to the formation of iron species such as Fe_3_O_4_ or Fe_2_O_3_ at the shell of the nZVI core [34]. This phenomenon is corroborated by the first-order derivative curve (see Fig. 6b).

### Structural and surface properties of PVDF-nZVI membranes

**Fig. 7:**
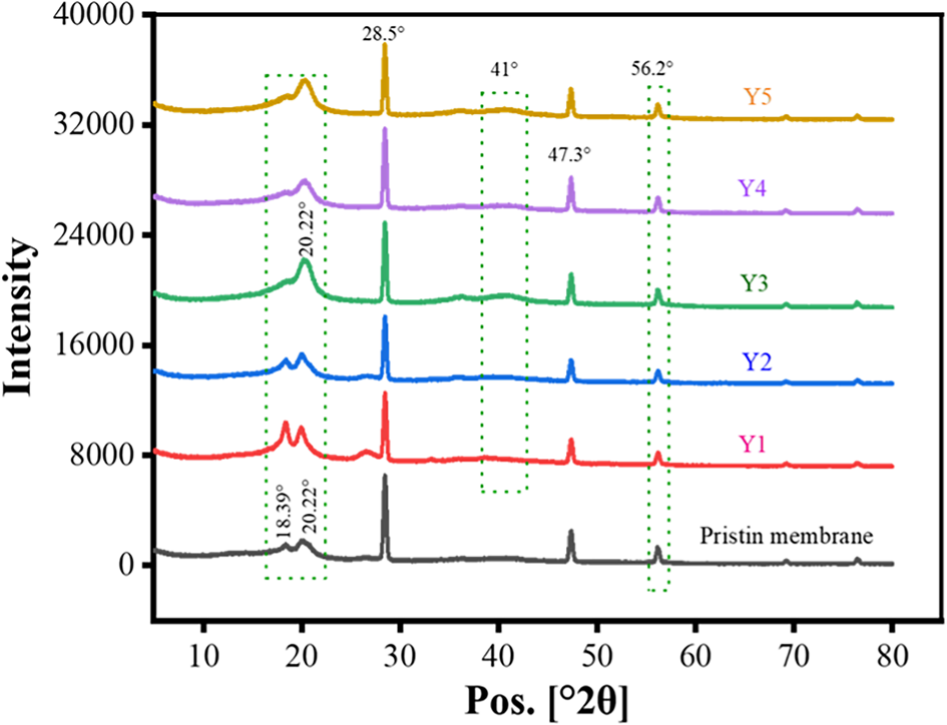
XRD patterns of pristin PVDF (Y0) and PVDF-nZVI nanocomposite membranes with varying nZVI loadings (Y1–Y5)

The incorporation of nZVI significantly influenced the structural and surface properties of the PVDF membranes. The XRD patterns (Fig. 7) showed clear peaks for both the α-phase and β-phase PVDF in all samples. In particular, the Y0 membrane shows a strong peak near 18.39°, which corresponds to the (110) plane of the α-phase, echoing findings from earlier research that identify this as a marker of pure PVDF’s crystalline form [27, 78, 79]. With the addition of nZVI nanoparticles (Y1–Y5), the diffractograms show marked changes, especially in the β-phase peaks, highlighting the substantial impact of nZVI on the polymer’s crystalline framework. Greater nZVI loading leads to a higher-intensity 20.22° peak, linked to the electroactive β-phase and signaling a shift toward a more stable β-phase at higher nanoparticle amounts. Further supporting this phase transformation is the development of additional reflections at 41°. These observations confirm previous studies, demonstrating that adding nanoparticles can improve the crystallinity and molecular organization of the polymer matrix, with the effect most pronounced at higher nZVI concentrations.

**Fig. 8:**
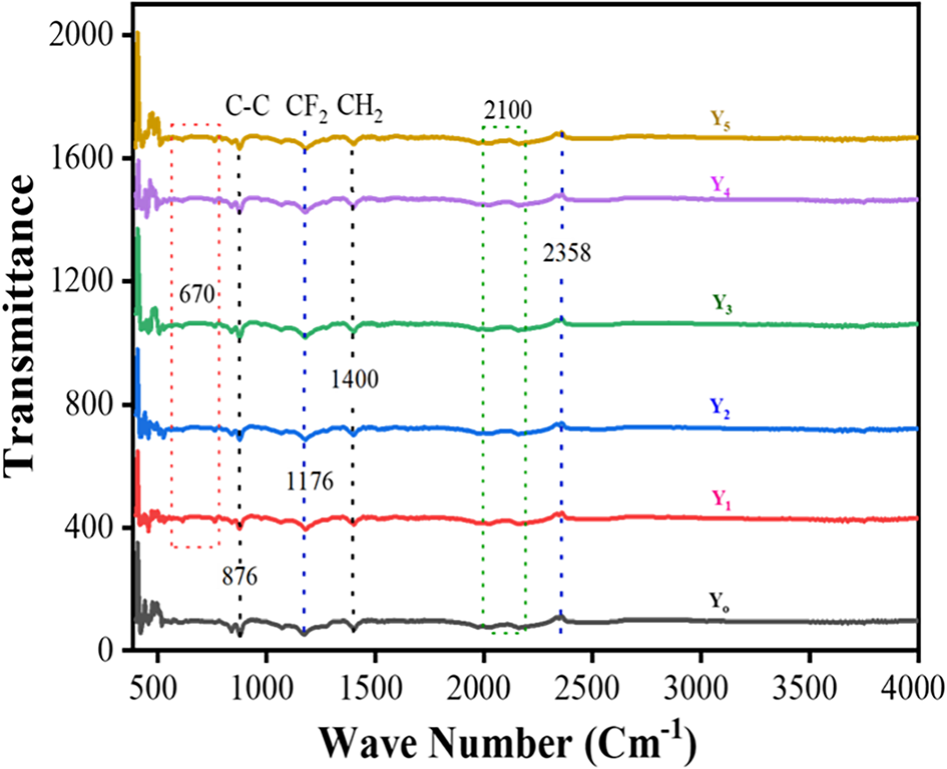
FTIR spectra comparing pristin PVDF (Y0) and PVDF-nZVI nanocomposite membranes (Y1–Y5)

FTIR analysis of PVDF-nZVI membranes (Fig. 8) examined interactions between nZVI nanoparticles and membrane functional groups to identify structural changes. The spectra for Y0 and the modified membranes (Y1–Y5) show that PVDF’s core chemical structure remains, though subtle spectral shifts occur with higher nZVI loadings. Characteristic peaks—such as CH_2_ wagging (1400 cm^–1^), CF_2_ asymmetric stretching (1,176 cm^–1^), C–C asymmetrical stretching (876 cm^–1^), and others—are consistent with prior studies [6, 11, 58, 74]. Increased peak intensities correspond to increased nZVI, while –OH stretching at 3,500 cm^–1^ supports previous findings [77]. Notable spectral differences between 670 and 876 cm^–1^ in the modified membranes suggest molecular rearrangements due to nZVI addition. These alterations imply potential PVDF restructuring and Fe–C carbide formation [69], as supported by X-ray diffraction and the emergence of M–O–H bands near 1250 cm^–1^, indicating complex interactions with increasing nZVI content.

FESEM analysis (Table 3) shows that nanoparticle dispersion within the PVDF membrane matrix remains consistent at lower loadings (Y1 to Y4). When nZVI nanoparticles are incorporated at concentrations between 0.1 g and 0.20 g, the nanocomposite membranes exhibit uniform distribution, indicating successful integration into the polymer matrix. In contrast, higher nZVI loadings (starting at 0.25 g) lead to notable agglomeration, consistent with previously reported findings on nZVI and titania-nanocomposite PVDF membranes [17]. These outcomes highlight how the intrinsic properties of nZVI influence their dispersibility in PVDF. XRD analysis further supports the observed morphologies at low nZVI concentrations, revealing a steady distribution of nZVI particles that aligns with earlier studies [52]. Effective nZVI dispersion at these lower concentrations may enhance membrane structural integrity. Agglomeration at higher concentrations primarily arises from strong interparticle forces—electrostatic, steric, and van der Waals interactions [3, 8].—which disturb the even distribution of particles in the matrix. The FESEM analysis (Table 3) shows that introducing nZVI into PVDF results in concentration-dependent changes in membrane structure. Membranes with 0.10–0.20 g nZVI show an improved surface pore profile, including larger mean pore sizes and increased overall porosity. Existing research confirms that controlling nanoparticle concentration can optimise these morphological features [1, 73]. Cross-sectional analysis highlights how varying nZVI amounts affect membrane morphology, particularly by creating extended macro-voids, a feature also observed in solution-cast membranes [35]. The formation of macro-voids is consistent with other studies [28, 38, 56], and relates to nZVI’s role in enhancing phase inversion. The addition of nZVI likely promotes earlier demixing by acting as nucleation sites or enhancing thermodynamic instability during phase inversion, resulting in a more compact structure with smaller surface pores (Table 4). Structural shifts driven by thermodynamic factors produce membranes with moderate porosity and selective layers. These altered structures are likely to increase water permeability [25, 71], yet the less compact morphology could reduce rejection performance and impair contaminant separation efficiency.

**Table 3:**
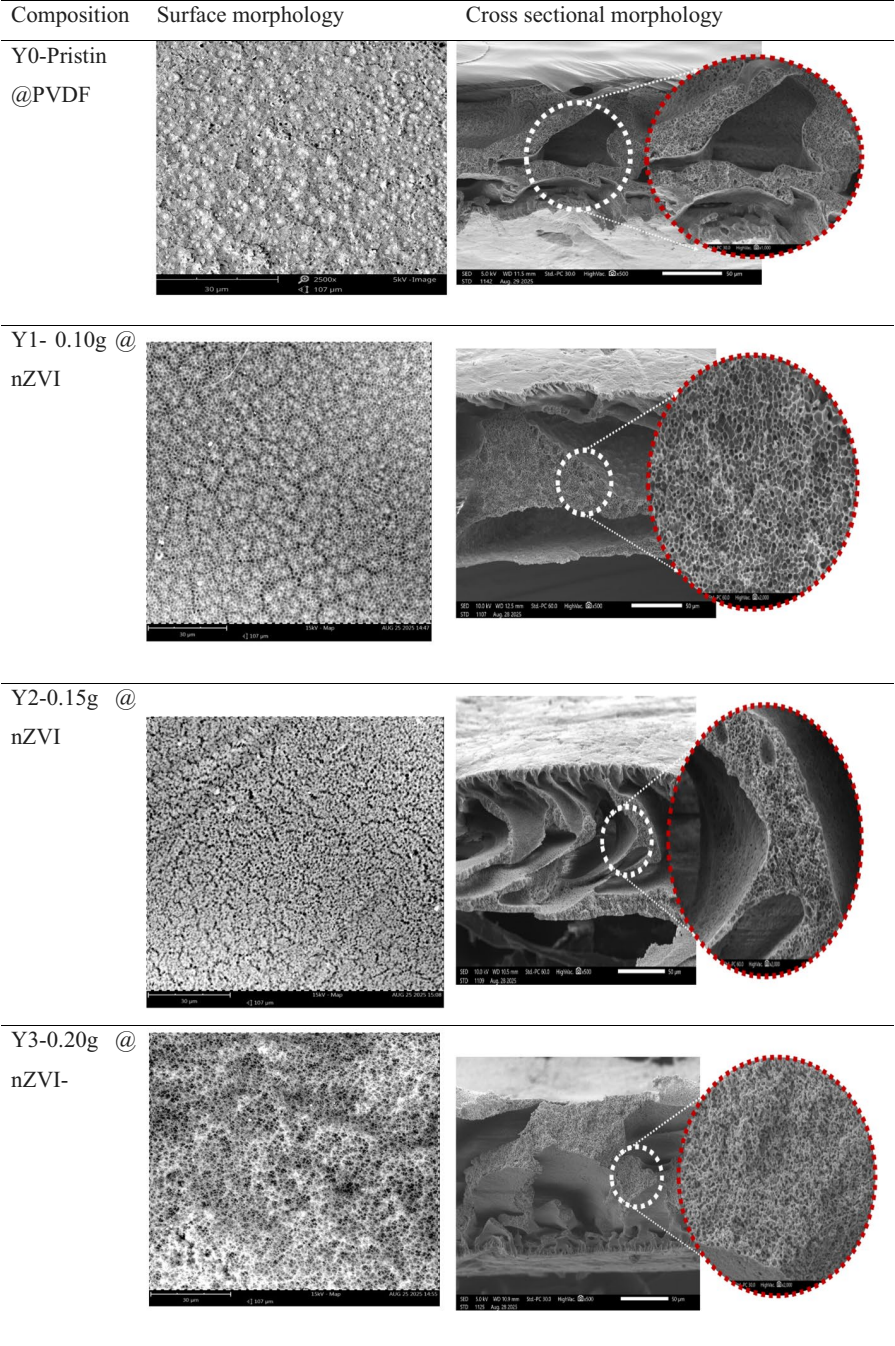

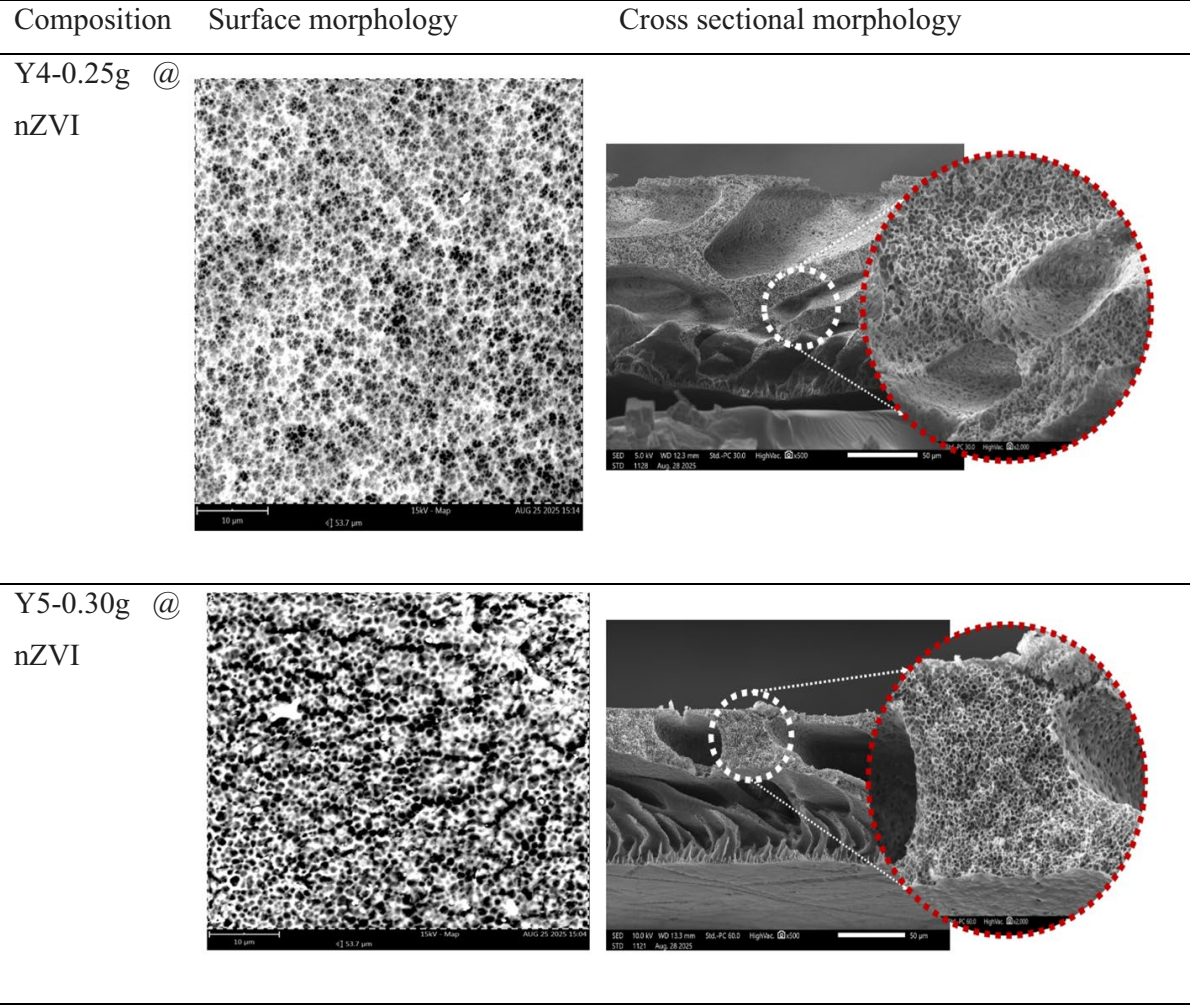
Surface and cross-sectional morphological characteristics of pristin PVDF and PVDF-nZVI nanocomposite membranes

**Table 4:** Physicochemical properties (thickness, pore size, and porosity) of the fabricated PVDF-nZVI nanocomposite membranes

Modified membrane	Average membrane thickness (µm)	Membrane pore size (× 10^–3^ µm)	Membrane porosity (%)
0.10 g nZVi	43	11.874	78.51
0.15 g nZVi	43.8	11.836	79.80
0.20 g nZVI	43.4	11.747	89.41
0.25 g nZVI	43.6	11.770	88.10
0.30 g nZVI	45.7	11. 746	83.16

**Fig. 9:**
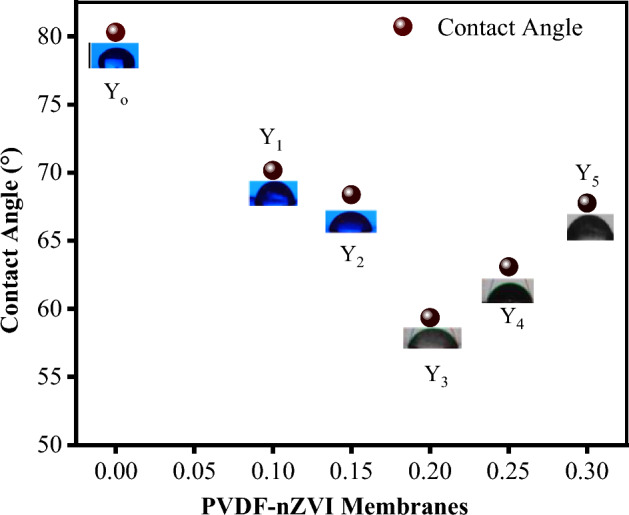
Water contact angle measurements assessing the surface hydrophilicity of PVDF-nZVI membranes at varying nanoparticle loadings

Hydrophilicity, or the membrane’s affinity for polar solvents such as water, is critical for environmental and water-purification applications [59, 60]. Water contact angle measurements (Fig. 9) show that increasing nZVI loading improves surface hydrophilicity due to the iron shell’s properties, with pristin PVDF showing a hydrophobic angle of 80.29°, decreasing to an optimal 59.35° at 0.2 g loading. Further increases in nZVI cause contact angles to rise again, indicating reduced hydrophilicity likely caused by nanoparticle agglomeration, which decreases membrane performance [31].

**Fig. 10:**
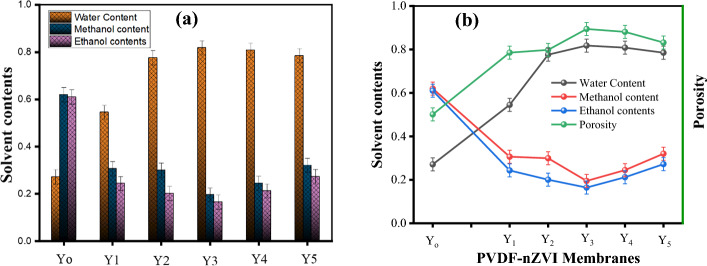
Solvent interaction analysis: **a** Equilibrium solvent content for water, methanol, and ethanol; **b** Correlation between solvent content and membrane porosity across membrane configurations

The solvent content of PVDF-nZVI membranes was tested with water, methanol, and ethanol and compared to membrane porosity (Fig. 10). The ineterpretation of these investigations are based on membrane surface swelling which are subsequently related with the corresponding membrane porosity. Methanol and ethanol showed an inverse relationship with porosity and hydrophilicity, consistent with previous studies [50, 51]. Water content increased from 0.55 to 0.78 as porosity rose from Y1 to Y5, indicating that more nZVI particles led to greater hydrophilic pores. The reduction in ethanol and methanol is due to selectivity, solvent polarity, and chemical interactions between modified PVDF and these solvents, highlighting the impact of nanoparticle loading and solvent-membrane interactions. The observed reduction in the solvent uptake could be attributed to differences in Hansen solubility parameters between the solvents and the modified PVDF matrix,as nanoparticle loading increases, the membrane’s effective solubility parameter shifts, reducing affinity for polar protic solvents while maintaining compatibility with water. This behavior is significant for water treatment applications because it indicates enhanced membrane stability against organic solvents that may be present in industrial wastewater, thereby reducing swelling-induced fouling or structural degradation.

### Hydraulic performance of PVDF-nZVI membranes

**Fig. 11:**
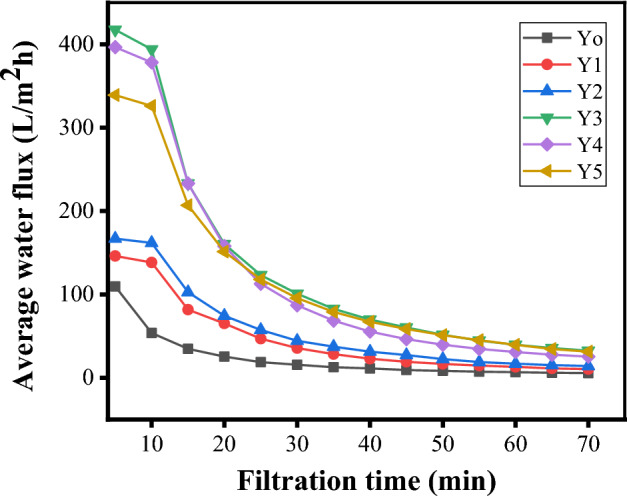
Pure water flux permeation profiles of pristin PVDF and PVDF-nZVI nanocomposite membranes over time

The hydraulic performance, measured via pure water flux (Fig. 11), showed a significant enhancement with nZVI incorporation. The unmodified PVDF (Y0) stabilized quickly, while membranes with higher nZVI nanocomposite loadings took longer—45 min for Y1, 50 for Y2, and 65 for Y3 to Y5. Steady-state flux increased with nZVI loading, reaching maximum values from 109.57 to 417.39 L m^−2^ h^−1^ across Y0 to Y4, but dropped slightly at the highest loading (Y5). This enhancement in flux performance correlates with lower contact angles, indicating improved membrane hydrophilicity. Notably, the data demonstrate that increasing nZVI content in the PVDF-nZVI matrix leads to superior pure water flux, consistent with findings from hydrophilicity assessments [53, 55] and solvent content analyses [50], as well as prior work highlighting the effect of hydrophilic properties on flux [21].

Beyond hydrophilicity, additional variables influencing pure water flux include membrane thickness, the formation of homogeneous interconnected pore networks, and potential agglomeration arising from surface modifications [72]. The observed decrease in flux performance after the 0.20 g nZVI loading (Y3) is likely attributable to elevated particle concentrations, which lead to pore blockage through agglomeration, thereby restricting water permeation. Moreover, recent research [80, 81] indicated that increased membrane thickness resulted in reduced pure water flux. However, morphological analyses with FESEM and supporting data from Table 4 indicate moderate structural characteristics and thickness levels, suggesting a limited impact of these factors on the overall flux performance of the nanocomposite membranes.

### Lead (Pb^2+^) removal performance

**Fig. 12:**
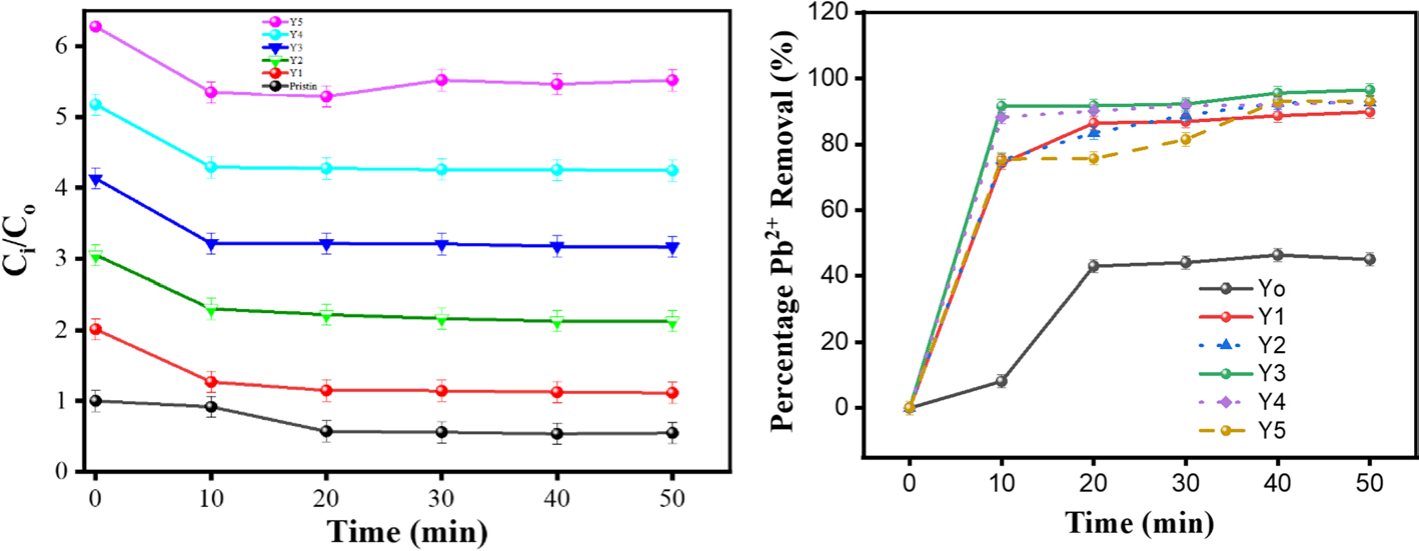
Pb^2+^ removal performance: **a** Concentration gradient profiles; and **b** Comparative removal efficiency (%) of membranes Y0–Y5 at 10 ppm feed concentration

To assess membrane performance and determine optimal PVDF-nZVI loading, filtration experiments were conducted using a 10 ppm Pb^2+^ solution. Results (Fig. 12a,b) showed a sharp initial decline in Pb^2+^ concentration across all membranes, could be attributed to the surface chemistry and charge effects rather than purely by physical adsorption as evidence by FTIR analysis [70]. The modified membranes (Y1–Y5) achieved removal efficiencies ranging from 89.8 to 96.54%, significantly surpassing that of the pristin membrane (Y0, 45.07%). Steady-state removal was established after 50 min, with the optimal loading (Y3) achieving 96.5% efficiency, likely due to increased active sites. However, further increases in nZVI loading led to agglomeration, reducing available surface area and removal efficiency, consistent with findings on nanoparticle overloading in similar systems [18, 44]. Notably, hydraulic properties were unaffected by agglomeration [43].

**Fig. 13:**
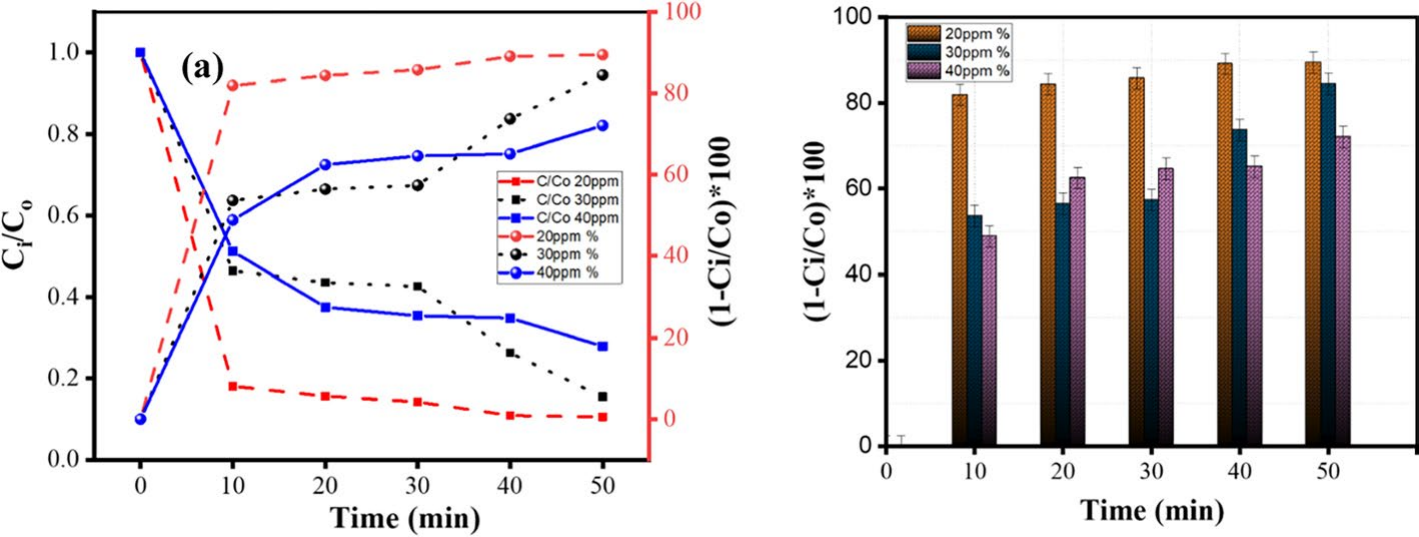
Performance of the optimal membrane (Y3) under varying initial Pb^2+^ concentrations: **a** Rejection efficiency profiles; and **b** Normalized filtration performance over time

The optimal membrane (Y3) was further tested with Pb^2+^ concentrations of 20, 30, and 40 ppm. Removal efficiencies declined with increasing contaminant concentrations (86.5%, 84.2%, and 75.9%, respectively; Fig. 13a), reflecting concentration-dependent performance [7] and possible active-site saturation [5, 30]. Rejection rates over time (Fig. 13b) remained consistent, with 80% removal achieved within 10 min at 20 ppm, though higher concentrations slowed reduction and separation due to site saturation. Despite this, the membrane maintained ≥ 75% efficiency across all tested concentrations, indicating robust long-term stability attributed to its hydrophilic properties.

**Fig. 14:**
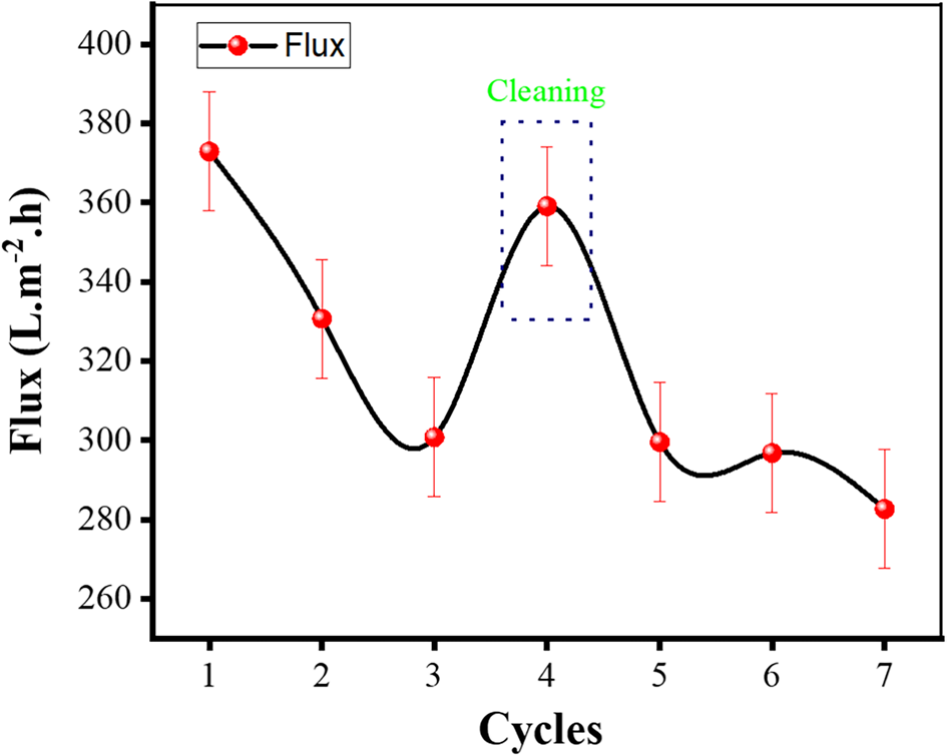
Flux analysis (5pmm Pb^2+^) of the optimal PVDF- nZVI modified nanocomposite membrane (Y3) over 7 successive filtration cycles

Figure 14 shows the operational stability of the optimal PVDF-nZVI nanocomposite membrane (Y3) following seven hours of filtration operation. The long-term operational stability and reusability of the optimal PVDF-nZVI nanocomposite membrane were assessed over seven consecutive filtration cycles, each lasting 60 min, using a 5 ppm Pb2^+^ solution (Fig. 14). A gradual decline in permeate flux was observed after the first two hours of cumulative operation, becoming more pronounced following three hours. This reduction in hydraulic performance is attributed to the accumulation of Pb2^+^ on the PVDF-nZVI surface. However, despite this trend, the observed flux decline was found to be reversible, as hydraulic cleaning effectively restored the original membrane flux stability.Subsequently, Table 5 compares the key metrics performance of the fabricated optimal PVDF-nZVI nanocomposite membrane with other nanocomposite membrane reported in the literature.

**Table 5:** Comparison of PVDF-nZVI key performance metrics with other nanocomposite membranes

Membrane material	Target contaminant	Removal efficiency	Water flux	Stability/Durability	Refs
PVDF-TiO2-nZVI	Pb2^+^	91% optimal removal of Pb2^+^	438.26 L m^−2^ h^−1^	Stable performance > 80% across all contaminant loadings	[45]
PVDF-TiO₂-nZVI-SiO₂	Pb2^+^, Cr (VI)	99.8% for Pb2^+^	563.47826 L m^−2^ h^−1^	Stable performance > 90% across all contaminant loadings	[45]
UiO-66-NH₂-g-DTPA/PES	Pb2^+^and Cu2^+^	99.79% (Cu2^+^)	6208 L·m^-2^·h^-1^	Stable at pH 3–10; remained high performance in cyclic test (6 cycles)	
1 T-MoS₂/PAN (TMP) with H₂O₂	Pb-EDTA complexes	97.37% for Pb-EDTA	Improved hydrophilicity	High performance over 5 cycles; processed ~ 300 L·m⁻2	[67]
PVDF-nZVI	Pb2^+^,	96.5% Pb2^+^	417.39 L m^−2^ h^−1^	96% Pb2 + removal while maintaining stable hydraulic properties	This work

## Conclusion

The current work systematically explored the synthesis, characterization, and performance assessment of polyvinylidene fluoride–nano zero-valent iron (PVDF-nZVI) nanocomposite membranes for the remediation of Pb^2+^ ions from aqueous media. The physicochemical properties of the synthesized nZVI and the resultant PVDF-nZVI composite were validated using comprehensive analytical techniques, including XPS and TGA, which confirmed the thermal stability and successful incorporation of nZVI nanoparticles. The fabricated PVDF-nZVI membranes exhibited notable improvements in hydrophilicity and maintained robust hydraulic properties throughout prolonged filtration operations. Optimal nanocomposite loading was identified, achieving a peak Pb^2+^ removal efficiency of 96%, while efficiencies consistently exceeded 75% across all tested membrane configurations and contaminant concentrations. These findings underscore the significant improvement in filtration performance and hydrophilicity imparted by nZVI integration into PVDF matrices, thereby advancing the efficacy of these membranes for practical water treatment applications. The Fe ions presence from the optimal membrane (Y3) was assessed after 5 h of filtration operation via AAS analysis. Although we have confirmed the presence of Fe ions in both the feed and the permeate, 0.211 ppm and 0.189 ppm respectively, considering the environmental standard on the iron content of 0.3 g/L (WHO/SDE/WSH/03.04/08) the optimal membrane possess no environmental concern. However, extended stability testing (8–12 h cycles) and more challenging fouling environments such as natural organic matter, competing ions (viz. Ca2^+^, Mg2^+^ and Na^+^), and real wastewater matrices are necessary to substantiate practical applicability.

## Data Availability

All data generated or analysed during this study are included in this published article.
